# The ER-Mitochondria Interface as a Dynamic Hub for T Cell Efficacy in Solid Tumors

**DOI:** 10.3389/fcell.2022.867341

**Published:** 2022-04-27

**Authors:** Elizabeth G. Hunt, Alex M. Andrews, Sydney R. Larsen, Jessica E. Thaxton

**Affiliations:** ^1^ Immunotherapy Program, Lineberger Comprehensive Cancer Center, University of North Carolina, Chapel Hill, NC, United States; ^2^ Department of Cell Biology and Physiology, School of Medicine, University of North Carolina, Chapel Hill, NC, United States; ^3^ Hollings Cancer Center, Charleston, SC, United States; ^4^ Department of Orthopedics and Physical Medicine, Medical University of South Carolina, Charleston, SC, United States; ^5^ Amherst College, Amherst, MA, United States

**Keywords:** endoplasmic recticulum (ER), ER stress, metabolism, cancer immunotherapy, T cell, tumor microenvironment, MERCs

## Abstract

The endoplasmic reticulum (ER) is a large continuous membranous organelle that plays a central role as the hub of protein and lipid synthesis while the mitochondria is the principal location for energy production. T cells are an immune subset exhibiting robust dependence on ER and mitochondrial function based on the need for protein synthesis and secretion and metabolic dexterity associated with foreign antigen recognition and cytotoxic effector response. Intimate connections exist at mitochondrial-ER contact sites (MERCs) that serve as the structural and biochemical platforms for cellular metabolic homeostasis through regulation of fission and fusion as well as glucose, Ca^2+^, and lipid exchange. Work in the tumor immunotherapy field indicates that the complex interplay of nutrient deprivation and tumor antigen stimulation in the tumor microenvironment places stress on the ER and mitochondria, causing dysfunction in organellar structure and loss of metabolic homeostasis. Here, we assess prior literature that establishes how the structural interface of these two organelles is impacted by the stress of solid tumors along with recent advances in the manipulation of organelle homeostasis at MERCs in T cells. These findings provide strong evidence for increased tumor immunity using unique therapeutic avenues that recharge cellular metabolic homeostasis in T cells.

## Primer: Immunotherapy and T Cell Biology

The adaptive immune system plays a substantial role in antitumor response with tumor infiltrating T lymphocytes (TILs) serving as the foundation for targeted cancer immunotherapies as they are responsible for penetrating tumors, recognizing tumor antigen, and directly killing malignant cells. High concentrations of CD8+ TILs are correlated with increased survival in multiple solid tumor types (Nakano et al., 2001; Sato et al., 2005; Sharma et al., 2007; Nguyen et al., 2016; Vihervuori et al., 2019), highlighting the critical role T cells play in cancer control. Due to diminished toxicity and robust response rates, development of immunotherapies utilizing TILs has swept the cancer care field. Initial immunotherapy efforts aimed to expand patient TILs *ex vivo* with high dose cytokines in order to infuse the cell product back to a donor, leading to increased tumor control in comparison to systemic treatment with IL-2 (Rosenberg et al., 1988). Since these initial studies, efforts in adoptive cellular therapy (ACT) have aimed to improve durability of the cellular product. Chimeric antigen receptor (CAR) T cell therapy improved upon traditional ACT by imbuing patient-derived T cells with specific, engineered antigen receptors that bypass the need for major histocompatiblity complex (MHC) presentation and coactivation while targeting molecules expressed on malignant cells, allowing T cells to more efficiently attack tumor (Mohanty et al., 2019).

Upon encounter with tumor antigen, both infused and endogenous tumor-specific T cells quickly exhibit exhaustion and undergo apoptosis, leading to loss of tumor control (Long et al., 2015; Davoodzadeh Gholami et al., 2017). Exhaustion is a phenotype defined by reduced proliferation and expression of inhibitory or checkpoint molecules marked by compromised cytotoxic cytokine production (Wherry and Kurachi, 2015). Programmed cell death protein 1 (PD1) is a specific checkpoint molecule enriched on antigen-specific T cells (Petrovas et al., 2006) that binds programmed cell death ligand 1 (PDL1) on tumor cells (Keir et al., 2008). Tumor cells expressing PDL1 evade the immune system through PD1-PDL1 ligation that leads to silenced effector cell function (Freeman et al., 2000) of the antigen-enriched TIL pool. Impairing PD1-PDL1 ligation through deletion of PD1 promotes clearance of tumors expressing PDL1 (Chen and Han, 2015) and monoclonal antibodies that target the interaction to overturn PDL1-mediated inhibition of TILs have revolutionized cancer care.

Naïve CD8+ T cells are exposed to tumor antigen *via* major histocompatibility complex I (MHCI) located on antigen-presenting cells (APCs) and subsequent costimulatory signals induce activation and clonal expansion, resulting in a pool of antigen-specific cells (van Stipdonk et al., 2001). Within the CD8+ TIL pool, subsets of effector and memory cells are capable of development, differing in terms of longevity of antigen response, cytotoxicity, and proliferative ability (Gerlach et al., 2010). The effector subset is highly cytotoxic with a short half-life and low capacity for long-term persistence, defined by interferon-γ (IFN-γ), granzyme B, and perforin secretion. Effector CD8+ T cells have high proliferative capability due in part to autocrine secretion of interleukin-2 (IL-2), but are short-lived upon re-exposure to antigen and subsequent cytolysis, dying through activation-induced cell death (Oshima et al., 1976; Smith et al., 1989; Schultz-Cherry et al., 2001; Joshi et al., 2007). From the initial phase of clonal expansion and contraction, a small pool of T cells survives – undergoing molecular changes to generate immunological memory, providing long-lived antigen recognition and rapid recall upon rechallenge (Opferman et al., 1999; Veiga-Fernandes et al., 2000; Kaech and Ahmed, 2001; Kaech et al., 2002).

A seminal discovery for the immunotherapy field was that central memory cells exhibit robust and durable tumor control, often promoting tumor clearance in comparison to effectors (Klebanoff et al., 2005). Similarly, infusion of memory T cells has proven beneficial for CAR T cell therapy, resulting in increased proliferation *in vivo* and vigorous tumor control (Gattinoni et al., 2005; Berger et al., 2008; Wang et al., 2012a; Sommermeyer et al., 2016). Effector and memory T cells are notably defined by their divergent metabolic programs. Prior to activation, naïve T cells exhibit low energy demand marked by high oxidative phosphorylation (OXPHOS) and fatty acid oxidation (FAO) (Pearce et al., 2013; Bantug et al., 2018b). Upon antigen exposure, differentiation into the effector state requires ATP for expansion, forcing a shift toward aerobic glycolysis (Wieman et al., 2007). In this state of metabolic activation, glucose and amino acid transporters are upregulated, glutaminolysis pathways engage, and FAO is suppressed by upregulation of transcription factor c-Myc (Frauwirth et al., 2002; Wang et al., 2011a; Sinclair et al., 2013). The memory cell metabolic profile opposes that of effectors in displaying sustained OXPHOS and FAO, with the majority of ATP content derived from mitochondrial metabolism (Pearce et al., 2009). Other studies indicate that a source of glucose for memory T cells is stored glycogen produced by an enhanced gluconeogenic cycle. Glycogen catabolism reinforces memory T cell antioxidant capability through generation of reduced glutathione equivalents that prolong survival while imbuing a cell intrinsic source of glucose (Ma et al., 2018).

The distinct metabolic programs of effector and memory cells are the likely cause for their differential antitumor efficacy. The tumor microenvironment (TME) presents a metabolic assault on CD8+ TILs including pH imbalance dysregulating metabolites, amino acid and glucose deprivation (Gajewski et al., 2013), and hypoxia (Petrova et al., 2018), all of which severely undermine the antitumor capability of effector TILs. For example, amino acid availability is crucial for T cell survival and metabolic fitness. Arginine deficiency in the TME compromises T cell tumor control, and L-arginine supplementation in T cells prompts a metabolic shift toward OXPHOS, resulting in therapeutic T cells (Geiger et al., 2016). Glucose deprivation in the TME similarly impairs gene expression events and cytotoxic functions in effector cells (Qiu et al., 2019), but metabolic remodeling of T cells away from glucose dependence drives memory formation and enhances T cell tumor control (Sukumar and Gattinoni, 2014). Also, a picture is emerging that effector cells are disproportionately affected by hypoxia as low oxygen drives abnormal proliferation, forcing increased glucose dependence (Xu et al., 2016) and enhancing terminal exhaustion in the endogenous CD8+ TIL pool (Yu et al., 2020). In contrast, the presence of memory T cells in the tumor bed is often linked to positive immunotherapy outcomes. For example, in the context of checkpoint therapies, memory markers on the TIL pool predict efficacy of treatment (Vanmeerbeek et al., 2021). In order to design successful novel immunotherapies, it is imperative to gain an understanding of the mechanisms through which memory TILs sustain function in the stress of the TME, resisting death, promoting long-lived tumor control.

### Tumor Stress Undermines Endoplasmic Reticulum Homeostasis in T Cells

The exquisite persistence of memory T cells in solid tumors is indicative of an intrinsic resistance to the metabolic stress presented by the TME. Perturbations to metabolic homeostasis such as amino acid deprivation, glucose starvation, and hypoxia promote the induction of the unfolded protein response (UPR) (Pan et al., 2003; Bi et al., 2005; Qiu et al., 2010; Bobak et al., 2016; Zanetti et al., 2022) in the endoplasmic reticulum (ER) of cells. As the hub of protein translation, the ER is equipped with three stress sensors, protein kinase RNA-like endoplasmic reticulum kinase (PERK), inositol-requiring enzyme 1 (IRE1α), and activating transcription factor 6 (ATF6) poised to sense a burden of unfolded/misfolded proteins in the ER lumen (ER stress) or other challenges to ER function such as lipid dysregulation (Hetz, 2012). The stress sensors initiate signaling pathways collectively referred to as the UPR that promote translational and transcriptional remodeling aimed to restore accurate protein synthesis and folding for re-establishment of cell homeostasis. Where ER stress pervades, pro-apoptotic signaling is enacted through the UPR, linking cell fate to duration and extent of ER stress (Cunha et al., 2012). Effector CD8+ TILs in mouse and human tumors experience ER stress mediated by PERK (Hurst et al., 2019a) and IRE1α (Song et al., 2018; Yu et al., 2020) and T cell-specific deletion of stress sensors contributes to improved tumor immunity. We have focused on the stress sensor PERK, demonstrating that memory T cells harbor diminished PERK protein relative to effectors (Hurst et al., 2019a) replete with robust reduction of the chronic PERK axis (Hurst et al., 2019a) characterized by transcription factors *Atf4, Ddit3* (C/EBP homologous protein (CHOP)) and their downstream transcriptional target *Ero1l* (Han and Kaufman, 2017). Similarly, the IRE1α-regulated transcription factor X-box-binding protein 1 (XBP1s) was found to contribute to terminal differentiation of antigen-specific T cells, reducing the formation of memory cells (Kamimura and Bevan, 2008). Together, the studies indicate that stress sensors may be differentially regulated among T cell lineages.

Diminished expression of stress sensors could serve to protect memory TILs from metabolic stress in the TME that engage the ER stress response, undermining CD8+ TIL antitumor function. In the TME, CD8+ TILs encounter glucose deprivation, a stress that immediately engages the UPR in glucose-dependent cells (Wengrod et al., 2015). In this context, PERK arrests global cap-dependent translation through phosphorylation of eIF2 alpha subunit (p-eIF2α) in an effort to restore cellular proteostasis (Harding et al., 1999). Our data indicate that translation attenuation in effector CD8+ TILs directed by robust upregulation of p-eIF2α undermines T cell ability to control tumor growth. In contrast, memory-like T cells express diminished p-eIF2α upon culture in TME conditions, sustaining protein translation and tumor control (Hurst et al., 2020; Riesenberg et al., 2022). In solid tumors, CD8+ TILs encounter lipid stress mediated by exposure to high levels of exogenous tumor-derived cholesterol. Exposure to cholesterol was found to induce terminal exhaustion in CD8+ TILs in a process linked to IRE1α activation. Inhibition of XBP1s diminished T cell exhaustion and augmented tumor control (Ma et al., 2019). In ovarian cancers, ascites fluid from ovarian cancer patients prohibited glucose uptake by T cells, ultimately leading to IRE1α-XBP1 activation that repressed mitochondrial respiration. IRE1α-XBP1 activation was found to prohibit the compensatory mechanism of glutamine uptake needed to maintain mitochondrial respiration in glucose deprived states. The study elegantly demonstrated that XBP1-deficient T cells enriched effector function in mouse models of ovarian cancer, providing evidence for the IRE1α axis as an immunotherapeutic target in cancer (Song et al., 2018). Together the data suggest that T cells endowed with mechanisms to support intrinsic resistance to TME metabolic stress exhibit heightened tumor killing capacity.

### Tumor Stress Impairs Mitochondrial Function in T Cells

Mitochondrial regulation has largely been the focus of CD8+ TIL immunometabolic study as the intratumoral subset exhibits severe alterations in mitochondrial morphology (Yu et al., 2020). T cells in solid tumors experience diminished mitochondrial biogenesis marked by reduced mitochondrial mass and hypopolarization. Mitochondrial biogenesis, growth, and replication of mitochondria increases mitochondrial mass and metabolic capacities that are crucial for T cell activation, proliferation, and ATP production (Desdin-Mico et al., 2018). Increased biogenesis through overexpression of PPAR-gamma coactivator 1α (PGC1α) enriches mitochondrial mass and supports T cell tumor control (Scharping et al., 2016). In line with these data, it was discovered that memory T cells have enriched mitochondrial mass relative to effectors, illustrating an intrinsic advantage for stress resistance in the TME (Buck et al., 2016). PD-1 (+) (exhausted) TILs display a reduction and shortening of mitochondrial cristae (Ogando et al., 2019) with disrupted membranes and low mitochondrial activity per unit of mitochondrial mass. Hypoxia was identified as a TME condition that represses PGC1α expression in PD-1 TILs as hypoxic exposure coupled with persistent antigen stimulation in the TME drove mitochondrial dysfunction marked by excessive mitochondrial reactive oxygen species (ROS), exacerbating the terminal exhaustion state (Yu et al., 2020). Thus, stimulation of mitochondria through ROS signaling or PGC1a activators augmented the efficacy of T cell response to a-PDL1 therapy (Chamoto et al., 2017). Together, these data illustrate gross dysregulation of mitochondrial biology in CD8+ T cells in tumors.

## Endoplasmic Reticulum-Mitochondria Interface Under Pressure

### Potential Impacts of Endoplasmic Reticulum-Mitochondrial Interaction in T Cells

The role of the ER in the paradigm of mitochondrial dysfunction in TILs has not been explored. However, studies are emerging that associate CD8+ TIL dysfunction with distress between the two organelles. RNA-seq indicated that exhausted CD8+ TILs experience heightened ER stress relative to other CD8+ TIL subsets, indicative of a profound misfolded protein burden in this T cell pool. Along with exacerbated ER stress, this TIL pool exhibits elevated alterations in mitochondrial morphology characterized by ineffective elimination of damaged mitochondria (mitophagy), abnormal mitochondrial membrane structure, and fewer, shorter cristae compared to autologous splenic T cells (Yu et al., 2020). The data corroborate our previous finding that severe (chronic) ER stress mediated by the PERK axis disproportionately affects exhausted CD8+ TILs, replete with mitochondria dysfunction marked by excessive ROS generation (Hurst et al., 2019a). Together, a picture is emerging that links ER stress to impaired TIL mitochondrial homeostasis, bringing into question the interaction between the two organelles in tumor immunity.

### Stress-Mediated Fission and Fusion at Mitochondrial-Endoplasmic Reticulum Contact Sites

**FIGURE 1:**
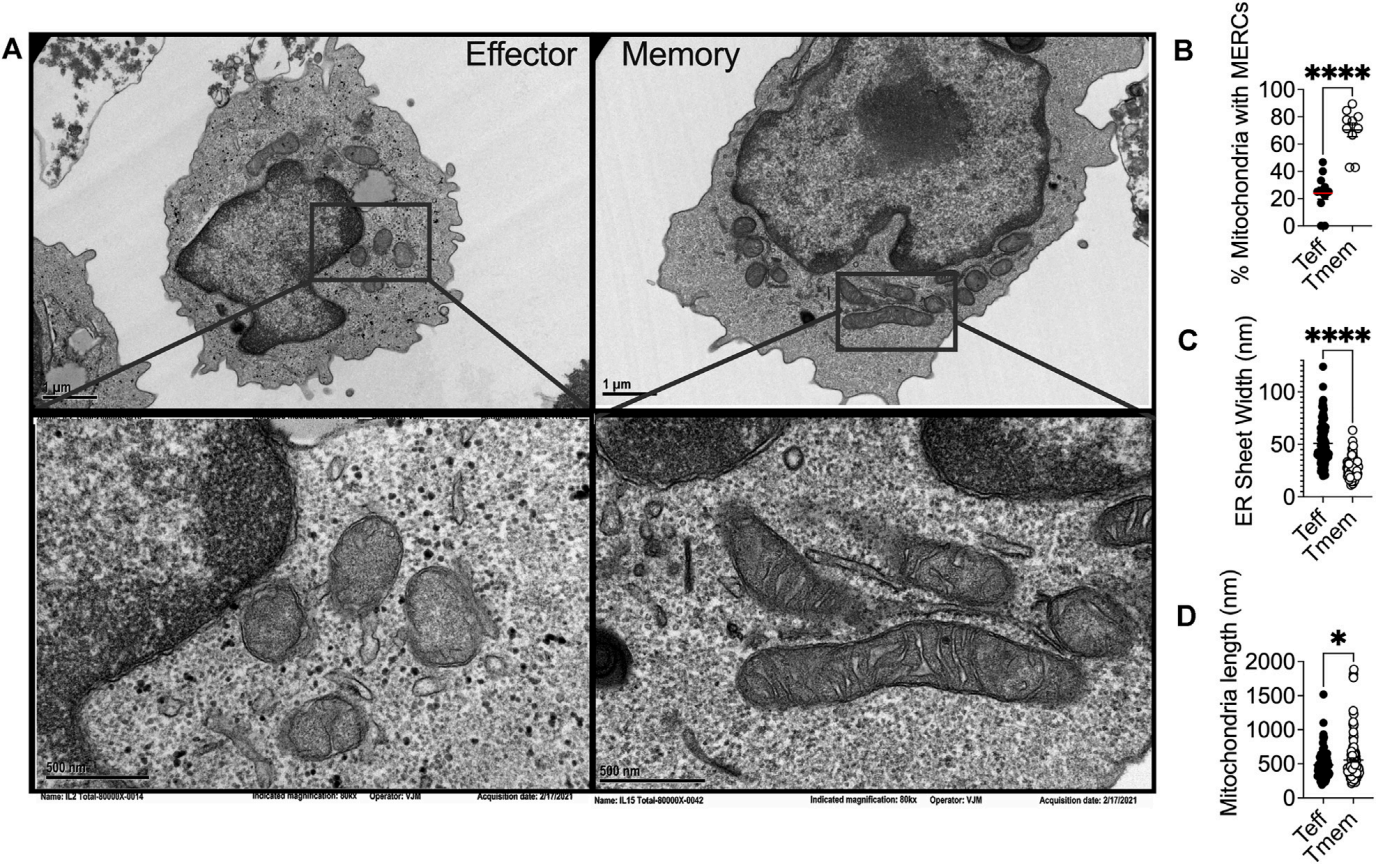
**(A)** Representative transmission electron microscopy of IL2 and IL15-derived effector and memory-like T cells, respectively, illustrating differential ER and mitochondrial biology in T cell subsets in alignment with previous reports that elucidated the phenomenon. *Ex vivo* expanded OT-1 T cells activated and expanded for 3 days with OVA peptide were conditioned for four more days in IL2 (200U) or IL15 (50 ng/ml) and **(B)** percentage of mitochondria possessing MERCs **(C)** ER luminal width and **(D)** mitochondrial length were quantified.

ER stress is associated with mitochondrial dysfunction in multiple disease pathologies (Giacomello and Pellegrini, 2016) and physical interactions between the ER and mitochondria often preclude organellar dysfunction. The ER and mitochondria are physically tethered at close distances of ∼10–25 nM (Giacomello and Pellegrini, 2016). These mitochondrial-ER contact sites (MERCs) are unique structures responsible for maintaining physical proximity and biomolecular signals between the two organelles. To this point, over 100 proteins have been identified at these sites as mediators of architecture or biochemical exchange, regulating fission, fusion, calcium (Ca^2+^), glucose, and lipid metabolism. MERCs may comprise up to 20% of the outer mitochondrial membrane (OMM) (Rizzuto et al., 1998). Though MERCs have not been studied in depth in CD8+TILs, transmission electron microscopy has been used to demonstrate that memory T cells display enhanced MERCs relative to effectors (Buck et al., 2016) (Figures 1A,B). These findings align with a study conducted on effector memory T cells where, upon activation, effector memory cells displayed increased MERCs compared to naïve counterparts, relying on contact sites to modulate mitochondrial function and rapid generation of IFN-γ (Bantug et al., 2018a).

### Endoplasmic Reticulum-Mediated Mitochondrial Fission

MERCs have been identified to play a role in regulating the dynamics of both the ER and mitochondria (Friedman et al., 2010; Friedman et al., 2011). Mitochondria undergo frequent fusion and fission to maintain organellar quality and cell homeostasis (Westermann, 2010) and the process is perceived to be regulated at MERCs. Mitochondrial fission is driven by the dynamin-related GTPase dynamin related protein 1 (Drp1), the fission protein which is recruited to the mitochondrial OMM and forms homo-oligomers around the mitochondria, constricting until one mitochondrion is severed in two (Macdonald et al., 2016). Live-cell imaging demonstrated that ER tubules mediate constriction of mitochondrial membranes, inducing fission in yeast and mammalian cells (Friedman et al., 2011). ER tubules physically wrap around mitochondria, forming MERCs, as the ER protein inverted formin 2 (INF2) recruits Drp1 from the cytosol to initiate fission. These data implicate MERCs and ER morphology in regulation of fission (Korobova et al., 2013) (Lee et al., 2016). Moreover, INF2 impacts ER-localized actin polymerization, enhancing MERCs formation, inter-organelle Ca^2+^ trafficking, and constriction of the inner mitochondrial membrane (IMM) (Chakrabarti et al., 2018). If MERCs are disrupted by ER tubule reorganization, mitochondrial fission and apoptotic signaling are similarly disturbed (Yedida et al., 2019). This regulation at MERCs may be reciprocal in nature, with disruption of Drp1 leading to disturbed ER morphology as well (Pitts et al., 1999; Westermann, 2011), as Drp1 overexpression can lead to both structural ER changes and mitochondrial fragmentation (Wikstrom et al., 2013; Kobayashi et al., 2020).

In yeast treated with ER stressors, both the ER and the mitochondria displayed expanded membranes prompting ER-mitochondria encounter structures and fission (Kojima et al., 2019). Upon induction of ER stress, the fission protein Drp1 is increased, resulting in mitochondrial fission and fragmentation (Li et al., 2015). ER stress increases MERCs formation, allowing for excessive Ca^2+^ transfer from the ER to mitochondria (Wanget al., 2021b), leading to mitochondrial fission and impaired bioenergetics (Kaddour-Djebbar et al., 2010; Glancy and Balaban, 2012). These data indicate the interconnectivity between chronic ER stress and mitochondrial fragmentation. ER stress can also induce apoptosis through the mitochondria, specifically through remodeling of cristae junctions for cytochrome c release (Zhang et al., 2008; Pinkaew et al., 2017). In response to ROS-induced ER stress, PERK stabilizes MERCs that relay ROS signals between the organelles, and sustains CHOP that induces mitochondrial apoptosis (Verfaillie et al., 2012). The data illuminate chronic ER stress as a driver of mitochondrial morphological changes, fission dynamics, and apoptotic signaling and could explain our previous finding that effector T cells with fragmented mitochondria express dramatically elevated PERK, failing to possess the resiliency and antitumor capabilities of their memory counterparts, expressing fused mitochondria (Figures 1A,D) (Buck et al., 2016) and diminished PERK (Hurst et al., 2019a).

### Endoplasmic Reticulum-Associated Mitochondrial Fusion

Differential mitochondrial biogenesis patterns impact T cell differentiation and immunotherapeutic efficacy. The major fusion proteins of mammalian mitochondria are the OMM GTPases mitofusin 1 and 2 (Mfn1/2) and the IMM GTPase optic atrophy 1 (OPA1) (Gao and Hu, 2021). These proteins serve to facilitate the two sequential steps of mitochondrial fusion at the OMM and IMM, respectfully. Mfn2 has been associated with MERCs formation and stabilization. Mfn2 deletion leads to decreased association between the ER and mitochondria, altering organellar morphology, reducing mitochondrial Ca^2+^ uptake (de Brito and Scorrano, 2008; Rizzuto et al., 2012). Similar to what has been observed in fission dynamics, the position at which mitochondria fusion occurs is marked by ER tubules (Abrisch et al., 2020), once again indicating that ER-mitochondrial interface acts on mitochondrial fission/fusion dynamics. Of note, memory T cells display fused mitochondrial networks (Figures 1A,D) with greater expression of Mfn2 and OPA1 (Buck et al., 2016). In contrast, effector cells require Drp1 for differentiation, with Drp1^−/−^ T cells displaying memory-like phenotype (Simula et al., 2018). Treatment of effector cells with fusion promoter M1 or Drp-1 inhibitor Mdivi-1 enforced fusion, leading to memory T cell development replete with heightened tumor immunity to murine lymphoma (Buck et al., 2016). Increased fusion is associated with organized cristae morphology (Varanita et al., 2015), enhanced oxidative phosphorylation (Rossignol et al., 2004; Simula et al., 2017), and increased mitochondrial mass (Buck et al., 2016), factors that contribute to enhanced persistence and tumor rejection by CD8+ TILs.

In contrast to chronic stress, acute stress in the ER followed by resolution of the unfolded protein burden promotes fusion of mitochondrial membranes. The PERK arm of the UPR is multifaceted in that it attenuates translation to resolve protein stress (Walter and Ron, 2011) and acutely serves to protect mitochondria homeostasis (Rainbolt et al., 2014). PERK localization to MERCs is required for maintenance of MERCs structure, with PERK^−/−^ mouse embryonic fibroblasts (MEFs) displaying decreased association between ER and mitochondrial proteins, fragmented ER morphology (Verfaillie et al., 2012), and fissed mitochondria (Lebeau et al., 2018). These data indicate that PERK plays a role in modulating mitochondrial fusion and morphology. PERK is critical to production of phosphatidic acid (PA) (Bobrovnikova-Marjon et al., 2012), a lipid responsible for facilitating fusion (Baba et al., 2014), potentially linking resolution of ER stress to mitochondria fusion dynamics through PERK. A direct correlation between PERK activation and mitochondrial dynamics was shown, with PERK-driven phosphorylation of eIF2α being required for mitochondrial hyperfusion in MEFs treated acutely (6–12 h) with ER stress enhancer Thapsigargin (Tg). However, longer treatments with Tg led to increased mitochondrial fragmentation, supporting the concept that chronic ER stress drives fission (Lebeau et al., 2018). Therefore, it is possible that among T cells, memory cells possess the ability to cope with and resolve ER stress, suggesting a cell intrinsic stress resistance that could explain sustained function in the TME.

### Stress-Induced Disruption of Endoplasmic Reticulum Dynamics

Disruption of ER structural homeostasis has been shown to directly impact mitochondrial morphology. The peripheral ER is a dynamic structure that interacts with and regulates numerous organelles including mitochondria and must be continuously remodeled to accommodate various demands such as protein production and folding. T cells are highly secretory and the rough ER acts as the primary site where secreted proteins are translated. Of similar importance in T cells, the smooth ER is the primary site of lipid synthesis (Higgins, 1974) and is the principal storage site for cellular Ca^2+^ ions (Schwarz and Blower, 2016). As a result of the diverse functions that the rough and smooth ER carry out, the ER is comprised of different sub-structures of sheets and tubules, respectively, that branch out to form a complex network consisting of the luminal compartment (Westrate et al., 2015). The distinct domains remain dynamic and undergo rapid changes (West et al., 2011) with the dense network of ER tubules extending to far edges of the cell enabling the ER to interact with other organelles including mitochondria.

In response to the stress of a protein burden in the ER lumen, the ER expands through proliferation of ER sheets to create increased volume for protein folding demand (Bernales et al., 2006). Cytoskeleton-linking membrane protein (Climp63) is the luminal protein that orchestrates ER expansion. Acting as a spacing protein, Climp63 determines the luminal width of ER sheets with depletion and overexpression resulting in decreased and increased luminal width, respectively (Voeltz et al., 2006; Shibata et al., 2010). In response to ER stress, Climp63 expression is upregulated coincident with UPR signaling (Schuck et al., 2009). Overexpression of Climp63 leads to impaired mitochondrial replication and reduced segregation of mtDNA during fission (Lewis et al., 2016). The impact of Climp63 on the mitochondria solidifies a connection between ER morphological proteins and mitochondrial dynamics and function. ER morphology has not been studied in T cell subsets, nor in the context of immunotherapy. Using transmission electron microscopy, we found that the ER of effector T cells displays expansion in comparison to that of memory cells, quantified by ER luminal width (Figures 1A,C). The data identify for the first time the possibility of differential ER morphology between T cell lineages and raise intrigue surrounding the potential impact of ER structure as a feature of T cell tumor immunity.

## Biochemical Exchange at Mitochondrial-Endoplasmic Reticulum Contact Sites: Metabolic Hubs

### Ca^2+^, A Matter of Life and Death

MERCs not only serve to shape structural outcomes between the ER and mitochondria, but are conduits for biochemical exchange, serving to facilitate cellular metabolic programming. Ca^2+^ is a key second messenger regulating apoptosis, exocytosis, and gene transcription (Clapham, 2007), whose exchange is centralized at MERCs. Ca^2+^ metabolism is paramount in T cells given that T cell receptor (TCR) engagement by antigen results in robust release of ER Ca^2+^ to tune the process of T cell activation and lineage fate. When the MHC-antigen complex interacts with the TCR, phospholipase C-gamma is phosphorylated leading to cleavage of phosphatidyl inositol, yielding diacylglycerol (DAG) and inositol 1,4,5-triphosphate (IP_3_) (Putney, 1986). Upon cleavage, IP_3_ binds IP_3_Rs, causing Ca^2+^ release from the ER lumen to cytosol, inducing nuclear factor of activated T-cells (NFAT) transcription (Fenninger and Jefferies, 2019; Hunt et al., 2020; Xiang et al., 2020), followed by IL2 production, T cell proliferation, and disease control (Klein-Hessling et al., 2017). The ER serves as the largest intracellular Ca^2+^ ion store, relying on Ca^2+^ signaling for normal protein folding and other homeostatic processes (Groenendyk et al., 2021). The ER Ca^2+^ gradient is dependent on import of Ca^2+^ from the cytosol into the ER lumen *via* sarcoendoplasmic reticulum Ca^2+^ ATPase pump (SERCA). In T cells, prolonged inhibition of SERCA through overexpression of the enzyme phosphoenolpyruvate carboxykinase 1 (Pck1) resulted in increased cytosolic Ca^2+^ that extended NFAT signaling, promoting effector function in tumors (Ho et al., 2015).

**FIGURE 2:**
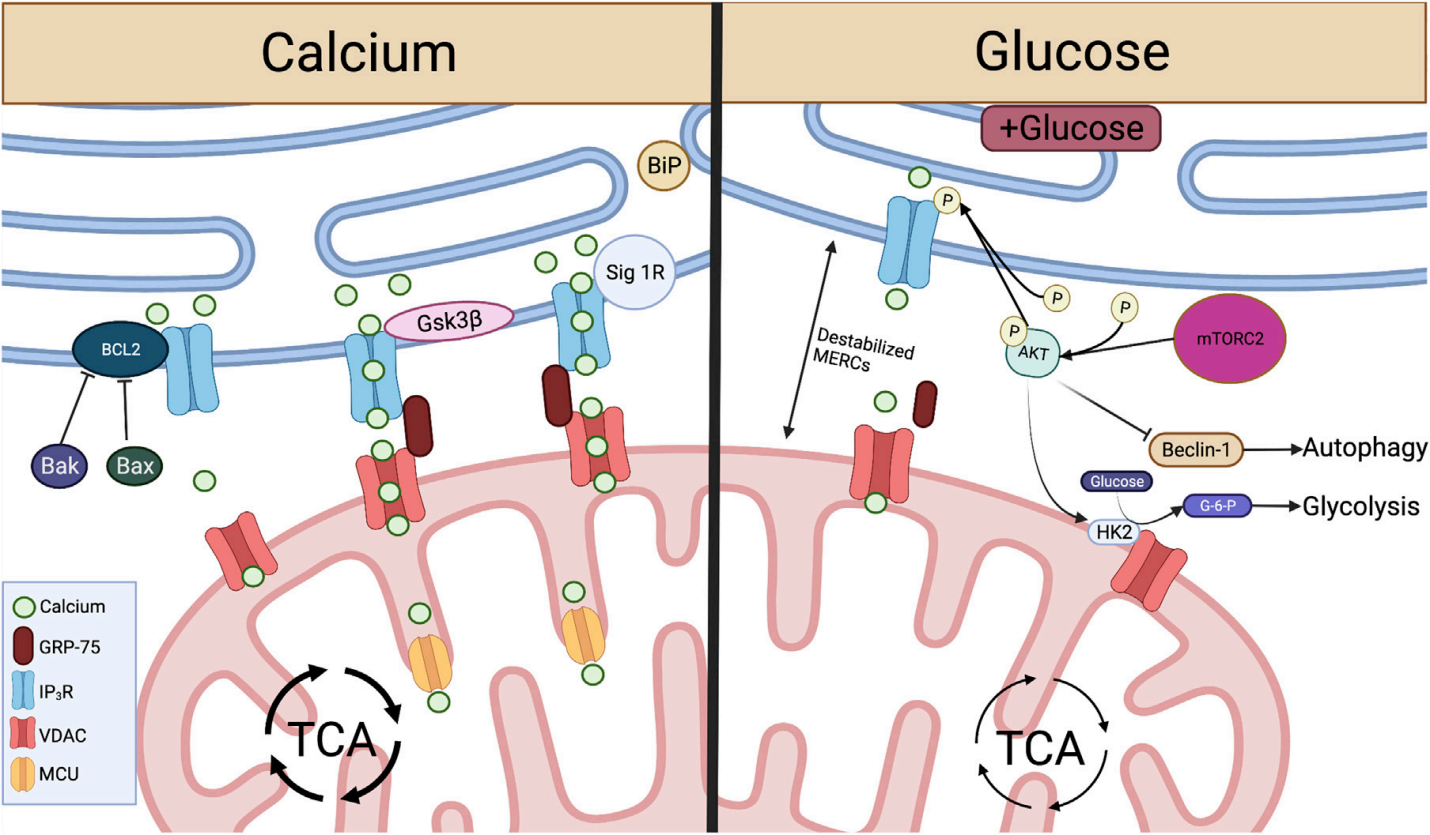
Crucial metabolite flow and nutrient sensing occurs at MERCs and is mediated by proteins acting on the IP_3_R-GRP75-VDAC complex. Left: At MERCs, Ca^2+^ is transferred from the ER lumen, through the IP_3_R-GRP75-VDAC complex, into the mitochondrial intermembrane space and through MCU to the mitochondrial matrix. This Ca^2+^ flux is promoted by Sig-1R dissociation from BiP and the protein Gsk3β interaction with IP_3_R. Ca^2+^ flux and IP_3_R-GRP75-VDAC association is inhibited by Bcl2, and this interaction is inhibited by pro-apoptotic proteins Bax and Bak. Right: When glucose is available, mTORC2 phosphorylation of AKT leads to reduced ER Ca^2+^ release and disruption of MERCs through inhibition of IP_3_R. pAKT also inhibits Beclin-1, a promoter of autophagy at MERCs. pAKT activation of HK2 leads to conversion of glucose to G-6-P, spurring glycolysis. Created with BioRender.com.

Specific microdomains known as “hotspots” have been identified where Ca^2+^ is concentrated around IP_3_R and mitochondria in close contact with the ER (Rizzuto et al., 1993), introducing the concept that transmission of Ca^2+^ between the ER and mitochondria occurs at MERCs. It was later demonstrated that interactions between the ER and mitochondria are regulated by the formation of a molecular bridge comprised of IP_3_R on the ER membrane, voltage dependent anion channels (VDACs) embedded in the OMM, and the chaperone 75-kDa Glucose Regulated Protein (GRP75), which facilitates the physical interaction between the two channels (Marchi et al., 2018). Upon IP_3_ binding, Ca^2+^ is released from IP_3_R at the ER and travels through the OMM into the mitochondrial intermembrane space by way of VDAC Ca^2+^ transfer. This interaction and subsequent Ca^2+^ transfer between IP_3_R and VDAC is mediated by appropriate MERCs formation (Honrath et al., 2017; Wang et al., 2021c). As Ca^2+^ enters the mitochondria and accumulates in the intermembrane space it passes through the mitochondrial Ca^2+^ uniporter (MCU) on the IMM to enter the matrix (Raffaello et al., 2016). There, Ca^2+^ activates dehydrogenases crucial for NADH production *via* the tricarboxylic acid (TCA) cycle (Figure 2), spurring mitochondrial respiration and ATP production (Vinnakota et al., 2016).

MERCs-facilitated Ca^2+^ flux from ER to mitochondria can be enhanced through direct protein-protein interactions with IP_3_R. Glycogen synthase kinase-3β (Gsk3β) is responsible for direct phosphorylation of IP_3_R, leading to increased Ca^2+^ transfer through the IP_3_R/GRP75/VDAC complex (Figure 2). However, over enrichment of this interaction drives cell susceptibility to apoptotic Ca^2+^ signaling (Gomez et al., 2016). In T cells targeting tumor, overactivity of Gsk3β has been linked to poor tumor control. Initial studies demonstrated that Gsk3β inhibition arrested T cell products prepared for adoptive cell therapy of melanomas in a stem-like state, enabling prolonged self-renewal and impairing differentiation (Gattinoni et al., 2009). The concept translated to the CAR T cell therapy field as Gsk3β inhibition diminished exhaustion hallmarks in CAR T cells transferred to glioblastomas, resulting in prolonged tumor control (Sengupta et al., 2018). Both studies identified a memory T cell phenotype in the T cell populations characterized by Bcl2^high^ expression, identifying a Ca^2+^ buffering effect by the antiapoptotic protein.

In contrast to enhanced Ca^2+^ flow from the ER to the mitochondria, the Bcl2 protein protects against mitochondria-mediated cell death by stabilizing Ca^2+^ flow through interactions with IP_3_R. Bcl2 is responsible for inhibition of excessive IP_3_R-mediated Ca^2+^ release, protecting cells from apoptotic Ca^2+^ signaling (Rong et al., 2008). Bcl2 also serves to protect cells from excessive ER Ca^2+^ buildup through enhancing IP_3_R sensitivity to low levels of IP_3_, leading to appropriate Ca^2+^ flow and increased mitochondrial bioenergetics (Eckenrode et al., 2010; White et al., 2005). Conversely, pro-apoptotic proteins Bax and Bak inhibit Bcl2-mediated IP_3_R stabilization, leading to reduced IP_3_R sensitivity and buildup of ER Ca^2+^, enabling robust Ca^2+^ release to the mitochondria, initiating programmed cell death (Oakes et al., 2005) (Figure 2).

The Bcl2 family of proteins are well-known for control of T cell antitumor efficacy. During development, Bcl2 expression is low in immature thymocytes. As T cells mature Bcl2 is increased as it is required for thymocytes to survive, protecting cells from programmed cell death (Shanmuganad et al., 2022). Across different T cell subsets, expression of Bcl2 family members differs, with naïve and memory CD8+ T cells expressing elevated Bcl2 in comparison to effectors (Grayson et al., 2000). Moreover, memory differentiation relies on Bcl2 expression as formation is impaired upon Bcl2 inhibition (Kurtulus et al., 2011). The data suggest that Bcl2 is a factor in long-term maintenance of immunity. Overexpression of Bcl2 in tumor-specific T cells promoted cell survival and therapeutic efficacy of adoptively transferred T cells infiltrating mouse melanomas (Charo et al., 2005; Kalbasi et al., 2010). Other studies of constitutive expression of Bcl2 in CAR-T cells resulted in prolonged T cell survival and tumor control in murine lymphomas (Wang et al., 2021a). Together, these data illustrate the importance of mediated Ca^2+^ flux by Bcl2 in T cell differentiation, longevity, and tumor control.

In addition to protein folding functions, ER chaperone proteins also play a role in control of Ca^2+^ flux and cell survival at MERCs. In a homeostatic state, the ER chaperone protein Sigma-1 Receptor (Sig-1R) localizes to MERCs and complexes with another chaperone, BiP (Grp78). ER Ca^2+^ depletion results in Sig-1R dissociation from BiP and subsequent interaction with IP_3_Rs, enabling prolonged influx of Ca^2+^ to the mitochondria. Therefore, overexpression of Sig-1R protects from cellular apoptosis, while depletion enhances cell death (Hayashi and Su, 2007) (Figure 2). These studies illustrate the role MERCs play as Ca^2+^ signaling hubs. Though knockout of IP_3_Rs leads to diminished ATP and loss of ER-mitochondrial contact maintenance (Cardenas et al., 2010; Bartok et al., 2019), we found that inhibition of IP_3_R in antigen-stimulated T cells promotes viability and memory formation. The resultant T cell pool showed enriched mitochondrial ATP reserves and robust capability to control murine melanomas (Thaxton et al., 2017), indicating that modulation of Ca^2+^ flux through IP_3_R tuning is intimately tied to T cell metabolism and tumor control.

### Glucose, Competing for Control

Ca^2+^ homeostasis is tightly connected to programming of glucose metabolism. Glucose is an essential nutrient for all mammalian cells, serving as a precursor for fatty acids, amino acids, and nucleotides (Navale and Paranjape, 2016). To sustain the energy demands of effector cells, CD8+ T cells shift metabolism from oxidative phosphorylation (OXPHOS) to aerobic glycolysis (Frauwirth et al., 2002; Jones and Thompson, 2007) and this is marked by an increase in glycolytic enzymes, lactate production, and glucose consumption (MacIver et al., 2013; Buck et al., 2015). Glucose is taken up directly by glucose transporters (GLUTS) that reside in the plasma membrane (Navale and Paranjape, 2016) and is phosphorylated by hexokinase 2 (HK2) to form glucose-6-phosphate (G-6-P) which is further broken down *via* glycolysis to pyruvate (Hay, 2016). In the cytosol, pyruvate can be converted to lactate or imported to the mitochondria through VDAC to fuel the Krebs cycle and electron transport chain. Mammalian target of rapamycin complex 1 (mTORC1) is the energy sensing complex that stimulates glycolysis, supporting protein synthesis and cell growth (Mao and Zhang, 2018). In T cells, deletion of negative mTORC1 regulator tuberous sclerosis complex 2 (TSC2) yielded highly differentiated hyper proliferative effector T cells with enhanced glycolytic dependence. T cells with TSC2 deletion resulted in a pool of robust antitumor T cells, but memory formation was impaired *in vivo* (Pollizzi et al., 2015). This study illustrated the importance of mTORC1 to produce the initial wave of effector response for tumor immunity, and the significance of mTORC1 to hinder memory cell metabolism and development of cellular longevity.

In response to glucose limitation, cells induce autophagy, a process by which cell components are catabolized to sustain cell function and viability (Yang and Klionsky, 2010; Dall'Armi et al., 2013). Autophagy has been shown to be critical for memory T cell formation (Xu et al., 2014). Active mTORC1 and glycolysis limit autophagy through disruption of a phosphorylation event carried out by energy deprivation sensor AMP-activated protein kinase (AMPK) (Kim et al., 2011), providing a mechanism to impede cellular degradation in nutrient replete settings. Upon induction of autophagy, a vesicular sac is formed enclosing cytoplasmic contents, resulting in a double membranous autophagosome that fuses with a lysosome to degrade contents (Lamb et al., 2013). Amino acids degraded from the process can be used to sustain translation and energy generation (Onodera and Ohsumi, 2005; Guo et al., 2016). A set of autophagy-related (Atg) proteins carries out the autophagic process and the protein light chain 3 (LC3) is widely observed on autophagosome membranes, thus this often serves as a ubiquitous marker for the process (Kabeya et al., 2000; Mizushima et al., 2004; Maruyama et al., 2014; Schaaf et al., 2016). During viral infection, autophagy was rapidly induced at the peak of effector T cell response, with virus-specific T cells exhibiting robust upregulation of LC3 proteins. T cell-specific gene deletion of the Atg7 or Atg5 proteins resulted in defective autophagy at the peak of effector response and impaired memory cell survival leading to faulty virus control (Xu et al., 2014). These data suggest that T cells with excessive mTORC1 activity and inability to form memory responses are likely defective in the initiation of autophagy due to abundant glycolysis.

MERCs play a dominant role in glucose sensing for cells to reprogram metabolism during fluctuations in nutrient availability (Tubbs et al., 2014; Theurey et al., 2016). MERCs were first implicated in nutrient-dependent autophagy by Hailey et al. (2010), who found that starvation-induced autophagy was inhibited after disruption of MERCs by Mfn2 deletion. Accumulation of autophagic proteins Atg14L and Atg5 and subsequent autophagosome formation occurred at MERCs in a starvation-dependent manner (Hamasaki et al., 2013). The process was found to be controlled by mammalian target of rapamycin complex 2 (mTORC2), a protein implicated in cell growth and metabolic function. Specifically, mTORC2-driven activation of the protein kinase AKT inhibits autophagy-promoting protein Beclin-1 (Wang et al., 2012b), resulting in suppression of autophagy. In nutrient deprivation, activation of AKT is inhibited (Betz et al., 2013), leading to induction of autophagy at MERCs through recruitment of Beclin-1 (Gelmetti et al., 2017).

MERCs were further linked to nutrient sensing when it was shown that hepatocytes of fasted mice display increased MERC lengths relative to fed counterparts (Sood et al., 2014). In contrast, hepatocytes from fed mice showed increased mitochondrial fragmentation as a result of decreased MERCs association through disrupted IP3R/VDAC1 interaction (Theurey et al., 2016). With increased pAKT and mTORC2 found at MERCs of fed mice (Betz et al., 2013; Tubbs et al., 2014), it has been hypothesized that this MERC disruption is a result of mTORC2 activation of AKT, resulting in inhibition of IP_3_R-VDAC interactions at MERCs (Szado et al., 2008; Betz et al., 2013). Glucose serves as the major determinant of these nutrient-dependent changes, with exposure to glucose disrupting MERC interaction both *in vitro* and *in vivo*, leading to increased mitochondrial fission and impaired respiration (Theurey et al., 2016). This can be attributed to MTORC2-AKT signaling, which promotes glucose uptake through activation of glucose transporters (Beg et al., 2017) and stimulates HK2-VDAC interactions, enabling conversion of glucose to G-6-P and therefore glycolysis (Stiles, 2009; Betz et al., 2013) (Figure 2). This AKT-dependent glycolytic switch is particularly important in memory T cells and has been shown to promote rapid effector function upon restimulation with antigen.

Two critical studies have offered insight to the necessity of autophagy in antitumor T cells. The studies draw divergent conclusions, highlighting the need for further study. DeVorkin et al. (2019) showed that T cell autophagy was disadvantageous to tumor control as mice deficient in autophagy genes Atg5, Atg14, and Atg16L1 displayed rejection of prostate, breast, and colorectal tumors. The effect was found to be immune-mediated based on diminished tumor growth in wild type mice reconstituted with Atg5^−/−^ bone marrow, and adoptively transferred tumor antigen-specific Atg5^−/−^ T cells demonstrated impaired antitumor immunity. Mechanistically, autophagy was responsible for metabolic dependence on OXPHOS; thus, limiting autophagy increased glycolysis and supported effector function—promoting tumor control (DeVorkin et al., 2019). This contrasts with a study conducted by Vodnala et al*.* (2019) T cell exposure to extracellular [K^+^], a cation enriched in the TME, limited T cell nutrient uptake and induced autophagy, diminishing acetylation of effector genes, promoting proliferation and self-renewal, indicative of stem-like T cells. K^+^-treated T cells were autophagy enriched and represented a pool of T cells with potent cytotoxicity and persistence in the TME that produced tumor clearance (Vodnala et al., 2019). However, the direct contribution of T cell-specific autophagy as a necessity or hindrance for rejection of solid tumors was not addressed. These studies illustrate the need to investigate the role of autophagy in CD8+ TIL metabolism and phenotypic modulation, as autophagy could serve as an avenue to enhance T cell-based immunotherapies.

\The balance between glucose dependence and autophagy that facilitates memory T cell development is an intriguing concept for understanding how to program effective T cell tumor immunity. On one hand hyperglycolytic T cells are powerful effectors for tumor control. On the other hand, T cells experience glucose deprivation in the TME that undermines their ability to persist as effectors (Ho et al., 2015). Interestingly, chronic low glucose conditioning (Klein Geltink et al., 2020) or long-term inhibition of glycolysis (Sukumar and Gattinoni, 2014) reprogrammed T cells toward a memory phenotype, likely as a result of autophagy induction. These data are intriguing in light of our recent discovery that acute glucose deprivation in T cells in the TME induces acute translation arrest through activation of the ER stress response (PERK-p-eIF2α), undermining sustained tumor control. We found that metabolic reprogramming of cells toward a memory lineage alleviated the stress response, abrogating p-eIF2α expression and restoring translation and tumor control to T cells in nutrient deprived environments (Riesenberg et al., 2022). The data raise the possibility that memory cells could use selective autophagy to eliminate stressed ER as a protective measure.

### Endoplasmic Reticulum-Associated Signaling and Structures in Autophagy

**FIGURE 3:**
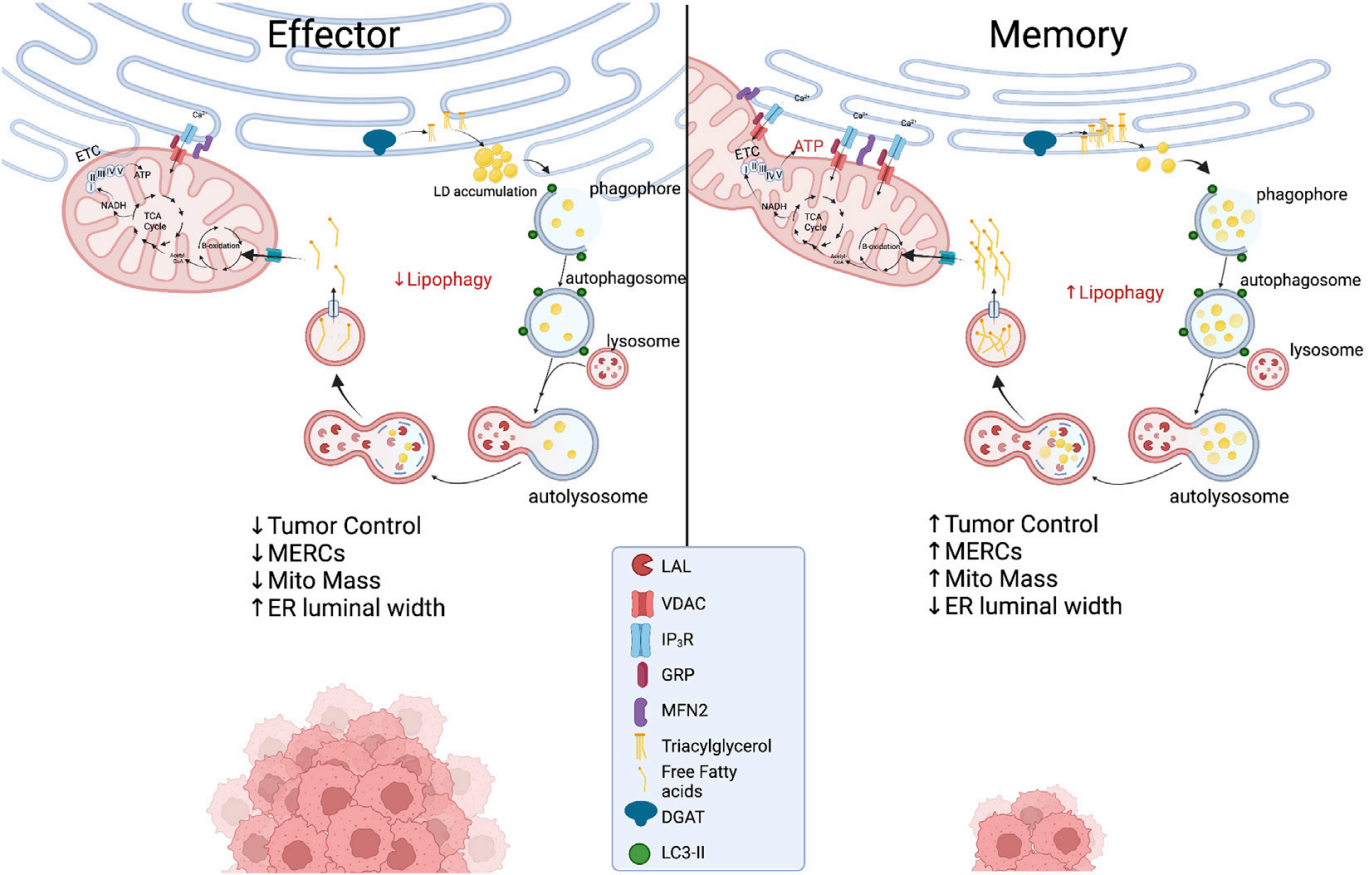
Proposed metabolic model at the ER-mitochondria interface in which effector T cells with expanded ER morphology experience chronic ER stress while displaying limited MERCs with punctate and fragmented mitochondria. In memory T cells, ER enzyme DGAT1 synthesizes triacylglycerides (TAGs) stored in lipid droplets that are concurrently degraded through lipophagy by LAL. The process could be accelerated in the nutrient stress of the tumor microenvironment, providing liberated free fatty acids to fused mitochondria to support energy generation in TILs. Created with BioRender.com.

The ER is a crucial organelle in the autophagic process. ER stress induces autophagy as treatment of cells with nutrient deprivation or ER stressor Tg forced expression of LC3 proteins and formation of autophagosomes. The process appears to be IRE1α-dependent as deletion of IREα leads to diminished autophagosome formation and impaired cell survival in response to ER stress (Ogata et al., 2006). This ER stress-mediated regulation of autophagy has not been well-studied in T cells, but IRE1α signaling has generally been found to be detrimental to T cell development and control of tumor growth. While ER stress instigates autophagy, structural entities derived in the ER are crucial components to orchestrate the autophagic response. Both omegasomes, a subdomain of the ER containing a lipid bilayer enriched for phosphatidylinositol 3-phosphate [PI(3)P], and ER-generated lipid droplets (LDs), are integral to initiation of autophagy. Premature phagophores that eventually mature into autophagosomes initiate from the omegasome and their formation accelerates with starvation (Lamb et al., 2013). LDs are organelles originating from ER membranes encapsulated by a phospholipid monolayer, comprised of a neutral lipid core containing triacylglycerols (TAGs) and cholesterol esters (Walther and Farese, 2012; Hashemi and Goodman, 2015; Olzmann and Carvalho, 2019). In this way, LDs are a stored resource of fatty acids (FAs), providing a lipid rich nutrient source that can be released to the mitochondria upon nutrient limitation (Yan et al., 2019). In response to autophagy induction, LD formation is accelerated in the ER and LDs can be found in close proximity to the mitochondria, acting as a buffer from chronic ER stress (Velazquez et al., 2016) and supporting FAO-mediated OXPHOS through MERCs (Saito et al., 2019) (Figure 3). In acute myeloid leukemia cells, disruption of MERCs through VDAC1 or Mfn2 depletion diminished lipolysis, autophagosome development, and OXPHOS, highlighting the importance of MERCs to regulate lipid metabolism to support autophagy and survival in cells adapting to stress.

### Endoplasmic Reticulum-Associated Signaling and Structures in Lipophagy

As previously discussed, MERCs and autophagy appear to be enriched in memory T cells. A link between ER and mitochondria lipolysis to support autophagy in T cells in tumor stress has not been investigated; however, using pharmacological inhibition, it was discovered that ER-resident diacylglycerol acyltransferases 1/2 (DGAT1/2) that catalyze TAG synthesis sustained FAO and OXPHOS in memory T cells (O'Sullivan et al., 2018). Moreover, overexpression of DGAT1 rescued immunological memory in T cells deficient in TAG synthesis (Cui et al., 2015). Given that LDs were not detected in memory cells, it was discovered that FAs were directly liberated from lysosomes, as the lysosomal acid lipase (LAL) was elevated in memory T cells relative to effectors, proving necessary for cell survival *in vivo* (O'Sullivan et al., 2018). Thus, it is likely that memory cells use lipophagy to access internal lipid stores and this aligns with their superior function and persistence in nutrient deplete settings. Together, the questions remain: Do memory T cells access ER-derived lipid stores to fuel FAO in the stress of solid tumors? If so, is the process dependent on lipophagy and can the process be facilitated through increased interactions at MERCs (Figure 3)? Understanding lipid dynamics could allow for successful design of potent immunotherapies.

### Endoplasmic Reticulum-Phagy

A final intriguing concept for tumor immunotherapy is that of ER-phagy, the orchestrated removal of damaged ER. In solid tumors mitophagy, the selective degradation of marred mitochondria, is impaired and results in persistence of dysmorphic mitochondria in CD8+ TILs. Similarly, cell stress conditions promote ER-stress induced autophagy as well as the process of selective ER-phagy. ER turnover is enabled by recruitment of autophagic machinery to sections of the ER exposed to extreme ER stress. Retreg1 (*Fam134b*) is among the most researched ER-phagy receptors and binds the LC3/GABARAP protein complex on the autophagosomal membrane mediated by a LC3-containing region (LIR) domain. In mice, disruption of *Fam134b* led to ER expansion, loss of ER turnover, and diminished ER stress resistance resulting in cell death (Khaminets et al., 2015). Nutrient deprivation induces ER degradation (Hamasaki et al., 2005) and in *Fam134b* competent MEFs, starvation induced degradation of Retreg1 was accompanied by loss of Climp63 expression. In contrast, in *Fam134b* ^−/−^ MEFs Climp63 expression remained elevated during nutrient starvation (Khaminets et al., 2015), indicating the paramount role of ER-phagy to promote regulation of ER dynamics in nutrient stress. These findings are intriguing, as they highlight the crucial process of maintaining ER homeostasis in cells through membrane remodeling, but this process has yet to be studied in the context of TILs or ER-mitochondrial interactions.

## Concluding Remarks

MERCs have emerged as regulatory hubs for maintenance of cellular function and survival. The contacts serve as regulatory points in eukaryotic cells, facilitating metabolite exchange and organelle morphology in order to promote cell survival, differentiation, and proliferation. The importance of MERCs is widely recognized in the fields of metabolism and cell biology, but the profound effects of ER-mitochondrial signaling in T cell differentiation and immune function in the suppressive TME are understudied. Our observations indicate that T cell subsets likely display altered ER morphology that could lead to differential MERC formation and mitochondrial morphology. The variability in MERCs likely impacts metabolic signaling, stress response, and autophagic patterns in T cells, ultimately facilitating the efficacy of an immune response in the TME. Now, we emphasize the need for more comprehensive studies on this topic to elucidate the role of ER-mitochondria interactions responding to cell stress, defining organelle structure, and function in nutrient deprivation in order to help bolster future T cell-based cancer immunotherapies.
